# Neurofeedback Training of Auditory Selective Attention Enhances Speech-In-Noise Perception

**DOI:** 10.3389/fnhum.2021.676992

**Published:** 2021-06-22

**Authors:** Subong Kim, Caroline Emory, Inyong Choi

**Affiliations:** ^1^Department of Speech, Language, and Hearing Sciences, Purdue University, West Lafayette, IN, United States; ^2^Department of Communication Sciences and Disorders, University of Iowa, Iowa City, IA, United States; ^3^Department of Otolaryngology – Head and Neck Surgery, University of Iowa Hospitals and Clinics, Iowa City, IA, United States

**Keywords:** selective attention, attentional modulation, neurofeedback training, perceptual training, speech-in-noise perception, eletroencephalography, brain-computer interface

## Abstract

Selective attention enhances cortical responses to attended sensory inputs while suppressing others, which can be an effective strategy for speech-in-noise (SiN) understanding. Emerging evidence exhibits a large variance in attentional control during SiN tasks, even among normal-hearing listeners. Yet whether training can enhance the efficacy of attentional control and, if so, whether the training effects can be transferred to performance on a SiN task has not been explicitly studied. Here, we introduce a neurofeedback training paradigm designed to reinforce the attentional modulation of auditory evoked responses. Young normal-hearing adults attended one of two competing speech streams consisting of five repeating words (“up”) in a straight rhythm spoken by a female speaker and four straight words (“down”) spoken by a male speaker. Our electroencephalography-based attention decoder classified every single trial using a template-matching method based on pre-defined patterns of cortical auditory responses elicited by either an “up” or “down” stream. The result of decoding was provided on the screen as online feedback. After four sessions of this neurofeedback training over 4 weeks, the subjects exhibited improved attentional modulation of evoked responses to the training stimuli as well as enhanced cortical responses to target speech and better performance during a post-training SiN task. Such training effects were not found in the Placebo Group that underwent similar attention training except that feedback was given only based on behavioral accuracy. These results indicate that the neurofeedback training may reinforce the strength of attentional modulation, which likely improves SiN understanding. Our finding suggests a potential rehabilitation strategy for SiN deficits.

## Introduction

Understanding speech in noise (SiN) is crucial for effective communication. It has been repeatedly reported that the ability to understand SiN differs dramatically, even across normal-hearing individuals (Kumar et al., 2007; Moore et al., 2013). One reason for poor SiN understanding could be the deteriorated selective attention (Bressler et al., 2017). Indeed, selective attention ability (both behavioral performance and the attentional modulation of cortical responses) shows large individual differences even among young normal-hearing listeners (Choi et al., 2014), which may correlate with SiN performance (Strait and Kraus, 2011). Our recent finding showed that the amplitude ratio of auditory-cortical responses to the target speech and noise during a SiN task correlated with behavioral SiN performance (Kim et al., 2021), indicating that attentional modulation on neural encoding of acoustic inputs in the auditory cortex (AC) would be a key neural mechanism for successful SiN understanding (Hillyard et al., 1973, 1998; Mesgarani and Chang, 2012; Carcea et al., 2017).

Conventional hearing remediations through amplification do not always improve SiN ability, even when equipped with noise reduction algorithms (Bentler et al., 2008) since SiN perception involves much beyond the detection of quiet sounds. Instead, perceptual training is often considered as a solution for SiN difficulties (Whitton et al., 2014, 2017). Perceptual training facilitates neural plasticity to improve listeners’ auditory and cognitive abilities by having a trainee engaged actively with a challenging sound that exploits perceptual or cognitive resources (Lawrence et al., 2018). Active engagement in training and repeated exposure to novel sound may induce anatomical and physiological changes that occur across existing neural pathways and even includes the budding of new connections, resulting in better auditory or cognitive function (Wall et al., 2002; Tremblay, 2007). However, a frequently reported problem of perceptual training is that the training effect does not generalize to other auditory stimuli not used for the training (Fiorentini and Berardi, 1981; Wright et al., 1997). This generalization problem leads us to consider training that *directly* improves a key strategy for the SiN understanding: a training that reinforces attentional modulation of auditory cortical responses. Theories of learning claim that the target of training is manipulated by rewarding; the determination of feedback (i.e., reward or punishment) must be based on the target training component (Goodman and Wood, 2004). Thus, to enhance attentional modulation of cortical responses, a training paradigm should provide feedback based on the strength of attentional modulation. While traditional perceptual training provides behavioral feedback at the end of a trial (Fiorentini and Berardi, 1981; Wright et al., 1997), we instead consider providing neurofeedback using brain-computer interfaces. The goal of neurofeedback training is that if subjects learn how to adapt neural activity consciously, it may result in specific patterns of neural activity that reach the pre-defined threshold level, followed by a reward to the subjects (Vernon et al., 2003; Ros et al., 2010).

Recent studies (Whitton et al., 2014, 2017) showed that training on extracting low-intensity signals from background noise and sustaining attention to the signals resulted in enhanced SiN performance, and it was transferrable to untrained stimuli. However, in these experiments, since both auditory segregation and selective attention processing were involved, it was challenging to isolate the training effects. How, then, can we design a perceptual training paradigm that aims to reinforce the attentional modulation of cortical activity solely? We developed a neurofeedback training paradigm that *explicitly* enhances the attentional modulation of cortical auditory evoked responses. Two speech streams, spoken by a female and male speaker, were played from different directions (left and right) with no physical overlap in time (i.e., no energetic masking) to maximize stream segregation, while subjects were instructed to attend to one of those streams. 64-channel electroencephalography (EEG) decoded auditory selective attention from single-trial EEG signals (Kerlin et al., 2010; Choi et al., 2013; O’Sullivan et al., 2015) throughout this auditory selective attention training, but only the Experimental Group subjects received visual feedback determined by the EEG-based attention decoder. To help rule out a placebo effect, a control group underwent a similar selective attention training but did not receive neurofeedback. Since the accuracy of attention decoding from single-trial EEG signals reflects the strength of attentional modulation on cortical auditory evoked responses (Choi et al., 2013), providing the result of EEG-based attention decoding as neurofeedback (Sherlin et al., 2011) may reinforce users’ attentional modulation of cortical responses.

The goal of the present study is to provide evidence to support the concept of auditory selective attention training through such an EEG-based neurofeedback paradigm and explore its efficacy for SiN understanding ability. We hypothesize that the neurofeedback attention training enhances the neural encoding of target speech (or the suppression of unattended noise or both) and SiN performance, showing this training effect’s generalizability.

## Materials and Methods

### Participants

Twenty normal-hearing, native speakers of American English were recruited for this study [mean age = 23.2 years; SD = 1.33 years; 6 (30%) male]. Upon agreeing to the study, subjects were randomly assigned to either the Experimental or the Placebo Group (i.e., single-blinded design). All subjects completed four consecutive weeks of 1-h-per-week training and pre- and post-training SiN tests at their first and last visits. We obtained written informed consent, and all work has been completed in accordance with the Code of Ethics of the World Medical Association (Declaration of Helsinki). All study procedures were reviewed and approved by the University of Iowa Institutional Review Board.

The sample size can be justified by a power analysis based on the effect size reported by previous perceptual-training studies, including Whitton et al. (2014), demonstrating a 10% improvement in SiN performance, which would be a clinically relevant difference. The variance in SiN performance was estimated from our previous study (Kim et al., 2021) that used the same SiN task with the same signal-to-noise ratio (SNR). With these estimations, the current study required ten subjects per group, assuming the significance level of 0.05 and power of 0.80. Similarly to Whitton et al. (2014), we chose 4-week training period (1) to guarantee overnight consolidation that has been claimed necessary in perceptual training (Fenn et al., 2003; Eisner and McQueen, 2006; Davis et al., 2009) and (2) to prevent learning and memory of speech stimuli that were used in pre- and post-training tests (Bentler, 2000; Bosshardt et al., 2005).

### Experimental Design and Procedures

#### Attention Training Procedure: Experimental Group

**FIGURE 1:**
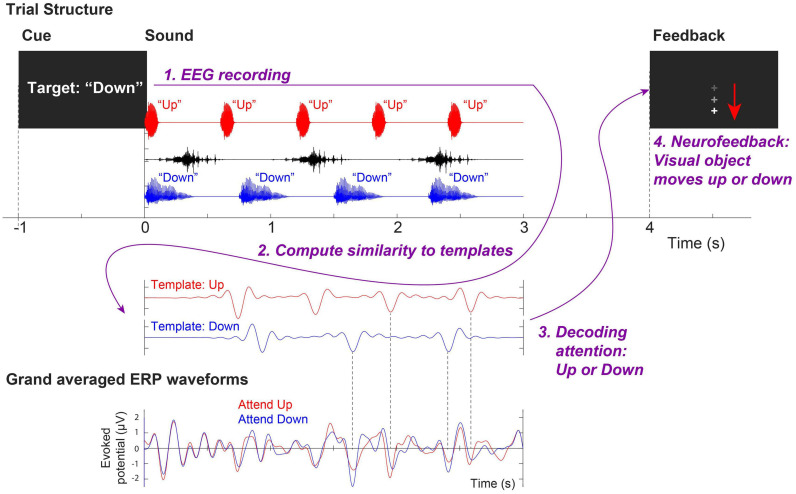
Trial structure of the neurofeedback training assigned to the Experimental Group. This example shows an attend-*down* trial.

During each training session, three overlapping auditory streams were presented; (1) a male voice saying the word “down” repeated four times from the right (+30° azimuth) loudspeaker, (2) a female voice saying the word “up” repeated five times from the left (−30°) loudspeaker, and (3) and a distractor non-speech noise that sounds like a water splash played three times intermittently from the loudspeaker directly in front of the subject. For each of the 120 trials in each visit, a visual cue (“Target: Up” or “Target: Down”) was given to direct participants’ attention to either the “up” or “down” stream (60 trials each). After the stimuli were presented, the attended stream was decoded from EEG. A visual feedback (“ + ” sign on the screen moving up or down) was given at the end of a trial to indicate the decoded direction of attention (i.e., attended “up” or “down” stream, respectively). Figure 1 illustrates an example of a trial attending the “down” stream.

#### Attention Training Procedure: Placebo Group

The Placebo Group listened to the similar three overlapping auditory streams (i.e., isochronous repetitions of “up” and “down” spoken by the female and male speakers with a distractor noise) where one of the last three (for “up” stream) or two (for “down” stream) utterances in each stream had three-semi-tone higher pitch. As the visual cue directed their attention to either the “up” or “down” stream in each trial, they picked an utterance with a higher pitch in the attended stream by pressing the number key (i.e., an oddball detection task within a trial). After the button press, visual feedback (“Correct” or “Incorrect”) was given based on the accuracy of their button response.

#### Pre- and Post-training SiN Tests

**FIGURE 2:**
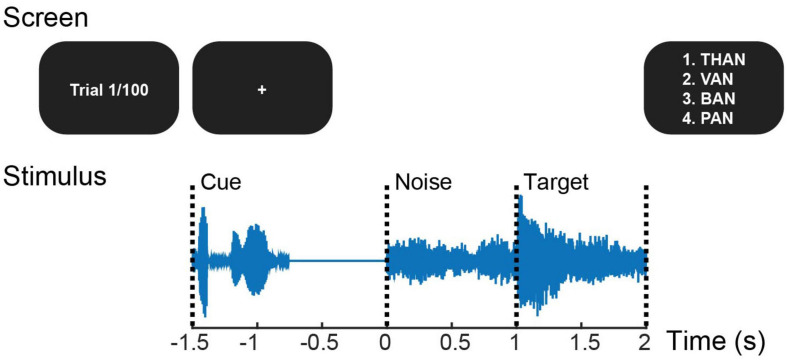
Trial structure of the pre- and post-training speech-in-noise task. At each trial that starts with the cue phrase “check the word,” a target word is presented a second after the noise onset. At the end of the trial, four choices are given, and the listener presses the button to answer.

All subjects, regardless of group designation, completed the same pre- and post-training SiN test (Figure 2) while EEG was recorded simultaneously. The test used 100 monosyllabic consonant-vowel-consonant English words from a pre-recorded California Consonant Test (Owens and Schubert, 1977) with added eight-talker babble noise. Stimuli were presented alternately at ± 3 dB SNR (50 words each) by changing the noise level in random order, while the target was presented at 65 dB SPL. At each trial, a target word started 1 s after the noise onset. At the end of a trial, subjects picked a word they heard from four choices that are fixed for each target word given on the screen. As in Kim et al. (2021) that calculated the amplitude ratio of auditory-cortical responses to target speech relative to noise, we placed the noise-only period before presenting the target speech to examine the training effect on cortical responses to target speech and ignored speech in parallel. Behavioral and neural data from the −3 dB SNR condition were only considered for analysis given the evidence that individual differences and cognitive effort could be maximized at the mid-point of SiN performance between 25% correct and 100% correct (Ohlenforst et al., 2017).

### Event-Related Potential Analysis

Sixty-four channel scalp EEG data were recorded during the training and SiN tasks using the BioSemi ActiveTwo system at a 2,048 Hz sampling rate with the international 10–20 configuration.

In order to provide neurofeedback to the Experimental Group, a template-matching method was used to decode the attended stream from single-trial EEG signals (Choi et al., 2013). EEG recordings from front-central channels (Fz, FCz, FC1, FC2, and Cz) were averaged and re-referenced to linked mastoids. EEG signals were bandpass-filtered between 1 and 9 Hz, baseline corrected, and then compared to two pre-generated template EEG waveforms obtained from grand-average cortical evoked responses to the single “up” and “down” streams during passive listening in quiet. The attention was decoded by finding the template that has a larger correlation coefficient with the single-trial EEG signal. Subsequently, the computer screen showed visual feedback accordingly by moving the fixation cross upward (indicating that attention to the “up” stream was detected) or downward (indicating that attention to the “down” stream was detected).

For analyzing EEG data obtained during SiN tasks, after applying a bandpass filter between 1 and 30 Hz using a 2,048-point FIR filter, epochs were extracted from −500 ms to 3 s relative to noise onset, baseline-corrected by using the average amplitude between −300 and 0 ms, and down-sampled to 256 Hz. Ocular artifacts (saccades and blink artifacts) were corrected by using independent component analysis (Jung et al., 2000). Since we use non-repeating naturally spoken words as stimuli, the latency of event-related potentials (ERPs) (e.g., N1) varied across words. To obtain clean N1 from averaged evoked response, every epoch was rearranged according to the median N1 latency of its corresponding word obtained from the grand mean of 50 normal-hearing subjects who completed the same SiN task in our laboratory previously. Then, the epochs were averaged at each electrode. The same preprocessing procedures were applied to both the Experimental and Placebo Group.

Since sensor data in a few channels may not adequately represent the spatial distribution of neural sources and temporal dynamics of ERP components (Tian and Huber, 2008), source localization needs to be applied to investigate temporal dynamics with the estimated source spatial distribution. In order to project the sensor-space data into source-space, the inverse operator was estimated using minimum norm estimation (MNE) (Hämäläinen and Sarvas, 1989; Gramfort et al., 2013, 2014) based on assumptions of multiple sparse priors (Friston et al., 2008) on an average template brain. Source-space time courses of ERPs were obtained across all cortical voxels in both hemispheres by applying the inverse operator. They were projected onto the cortical maps as a form of noise-normalization procedure providing dynamic statistical parametric maps (dSPMs) (Dale et al., 2000). A representative voxel for each cerebral hemisphere was chosen in the Heschl’s gyrus (HG), known to contain the primary AC (Da Costa et al., 2011), by conducting the cross-correlation analysis over time across voxels in HG and then selecting a voxel per hemisphere that showed the maximum average correlation coefficient (Tong et al., 2016). For statistical analysis, ERP data at the representative voxel were bandpass filtered from 4 to 8 Hz to capture auditory N1 and P2 components using a zero-phase 128-point FIR filter with symmetric non-causal impulse responses (de Cheveigne and Nelken, 2019). Then, temporal ERP envelopes were extracted by applying the Hilbert transform to the bandpass-filtered data and taking the absolute value. Obtaining ERP envelopes at the representative voxel in HG was to examine the effect of training in enhancing attentional modulation of AC responses by comparing ERP magnitude between conditions.

### Statistical Analysis

Two-way mixed ANOVAs were conducted on both behavioral performance and neural data. To investigate the training effect on AC responses, the peak magnitudes of ERP envelopes obtained at ∼230 ms after the noise/word onset were compared between conditions. We computed leave-one-out grand averages (i.e., jackknife approach) prior to testing to perform statistical analysis on neural data with taking advantage of getting clear ERPs from grand averages. Since we specifically hypothesized that only the Experimental Group (that received neurofeedback) would enhance the efficacy of attentional control through the neurofeedback training and that the training effect would generalize to an untrained SiN task, we decided to perform *post hoc* tests even when no significant global effect was found (Hsu, 1996). Also, a one-tailed paired *t*-test was chosen as the *post hoc* test since we were only interested in the improvement (i.e., one direction) in attentional modulation, SiN performance, and neural encoding. Bonferroni correction was applied to account for multiple comparisons across groups, training conditions (or training sessions), and two cerebral hemispheres. Inflated *F*-ratios and *t*-values due to the jackknife approach were adjusted (Luck, 2014).

## Results

### Changes in Attentional Modulation During Training

**FIGURE 3:**
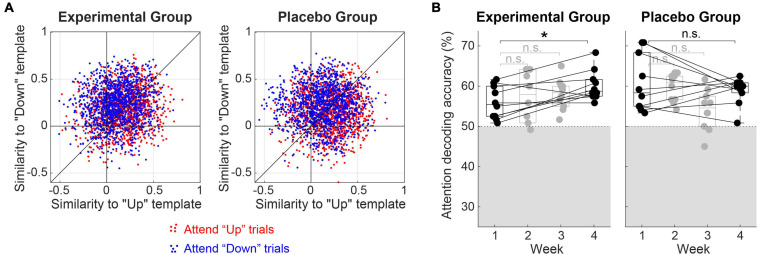
**(A)** Distributions of correlation coefficients (“Similarity”) between single-trial EEG waveforms and grand-average evoked responses to the single “Up” and “Down” streams (i.e., referred to as “Up” and “Down” templates). Red and blue dots represent trials from “Attend-Up” and “Attend-Down” conditions, respectively. The mean accuracy of attention decoding (i.e., calculated as the averaged ratio of blue dots above the diagonal and red dots below the diagonal) was 59.1% for the Experimental Group and 58.1% for the Placebo Group. **(B)** Changes in attentional modulation during training. Attention decoding accuracies from individual subjects are denoted as filled circles over 4 weeks of training. The gray shade indicates an area below the chance level (i.e., 50%). A box plot in each week shows the 75th percentile, median, and 25th percentile line. *Significant at *p* < 0.05, n.s.: not significant.

As described above, selective attention was decoded by comparing Pearson correlation coefficients between a single-trial EEG waveform and grand-average cortical evoked responses to the single “up” and “down” streams. The effect of attention on the single-trial EEG waveforms has been tested by counting the number of “Attend-Up” and “Attend-Down” trials that exhibited greater correlation coefficients with “Up” and “Down” templates, respectively, in each listener. Both Experimental and Placebo Groups showed a significant effect of attention (i.e., greater-than−50% accuracy from the single-trial counting) on EEG responses; one-sample *t*-test exhibited *t*_9_ = 8.34 (*p* < 0.001) for the Experimental Group and *t*_9_ = 5.12 (*p* < 0.001) for the Placebo Group. The mean accuracy of single-trial attention decoding (i.e., the ratio of single-trial EEG waveforms decoded as the correct direction of attention) across all the training sessions was 59.1 and 58.1%, with a standard deviation of 3.5 and 5.0% for the Experimental and Placebo Groups, respectively. Figure 3A shows the distribution of correlation coefficients across all the single trials from all the subjects in the Experimental (left panel) and Placebo (right panel) Groups. Red and blue dots represent “Attend-Up” and “Attend-Down” trials, respectively. There was no significant difference in the single-trial classification accuracies between the groups (two-sample *t*-test, *p* = 0.59). The mean accuracy of the oddball detection in the Placebo Group was 98.8%, with a 1.4% standard deviation.

Figure 3B shows the change in attentional modulation over time with repeated training. In the Experimental Group, the mean decoding accuracy increased monotonously from the first week’s 55.9% (SD = 4.1%) to 57.4% (2nd week, SD = 6.2%), 58.0% (3rd week, SD = 3.8%), and 60.2% (SD = 3.7%) in the last week. In contrast, the Placebo Group did not show improvement in attentional modulation over time. Mean decoding accuracies were 60.7, 59.7, 54.1, and 58.9% for the 1st, 2nd, 3rd, and the last week. Standard deviations were 7.0, 3.5, 5.0, and 3.4%, respectively.

To further investigate the effect of training time (i.e., first vs. fourth week), the type of feedback (i.e., neurofeedback vs. behavioral), and the interaction of those effects on the attentional modulation (i.e., quantified as the decoding accuracy), we conducted a two-way mixed ANOVA on the decoding accuracy observed in the first and last week. No significant main effects of time (*F*_1,18_ = 0.99, *p* = 0.33) and group (*F*_1,18_ = 1.0, *p* = 0.32) on the decoding accuracy were observed, indicating that (1) when combining both groups, there was no significant improvement in attentional modulation over time with repeated training and (2) there was no baseline-difference of attentional modulation between the groups. However, a significant interaction between time and group (*F*_1,18_ = 5.7, *p* = 0.028) was revealed, indicating that the training effect on attentional modulation over time significantly differed between the groups. The *post hoc* paired *t*-test between the first and the last weeks’ decoding accuracy exhibited a Bonferroni-corrected *p*-value of 0.022 (i.e., six times uncorrected *p*-value 0.0036) in the Experimental Group.

### Behavioral SiN Performance

**FIGURE 4:**
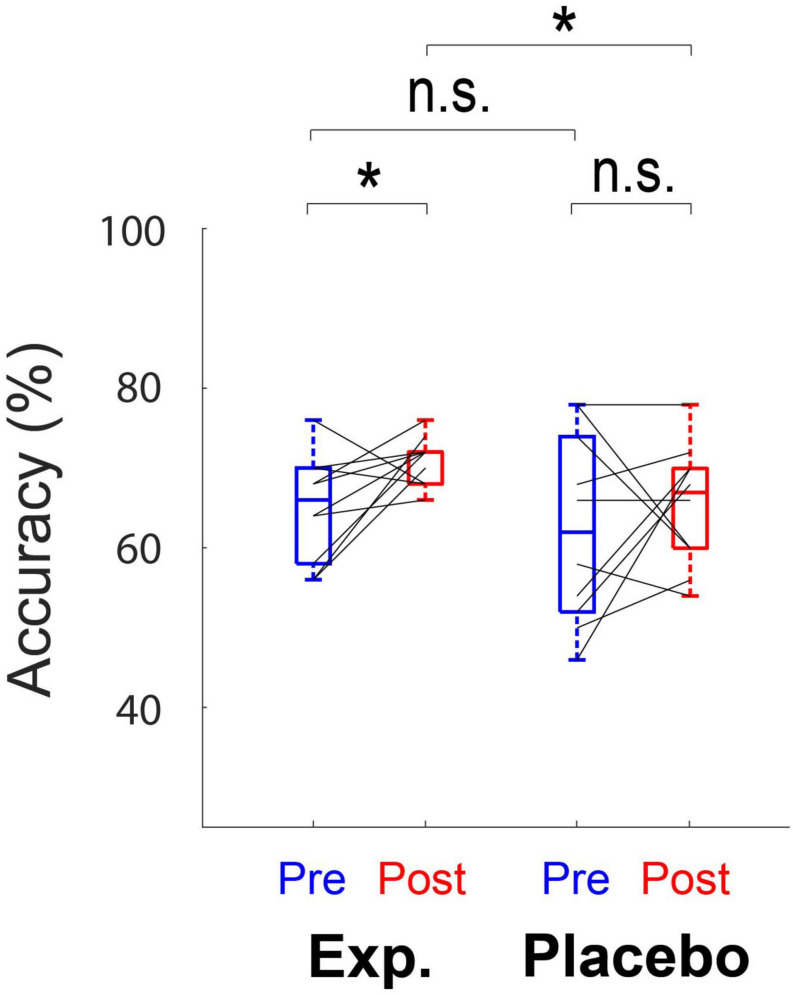
Box plots show the behavioral performance (accuracy) for both the Experimental Group and the Placebo Group. Each box denotes the 25–75th percentile range, and the horizontal bar in the center denotes the median. Solid lines indicate the same subject in different conditions. Mean accuracy significantly differs before and after training in the Experimental Group. *Significant at *p* < 0.05, n.s., not significant.

No significant main effects of training (*F*_1,18_ = 3.37, *p* = 0.083) and group (*F*_1,18_ = 2.49, *p* = 0.13) on behavioral performance (accuracy) were observed. No interaction between training and group (*F*_1,18_ = 0.38, *p* = 0.55) was revealed. Although the global effect of training and group was not significant, *post hoc* paired *t*-tests indicated that accuracy increased significantly in the Experimental Group (*t*_9_ = −2.37, adjusted *p* = 0.042), but not in the Placebo Group (*t*_9_ = −0.71, adjusted *p* = 0.49) (Figure 4). *Post hoc* analysis also showed a significant difference in the performance between two groups after training (*t*_18_ = 2.15, adjusted *p* = 0.045), but not before training (*t*_18_ = 0.60, adjusted *p* = 0.56) (Figure 4).

### Source-Space ERPs to SiN

**FIGURE 5:**
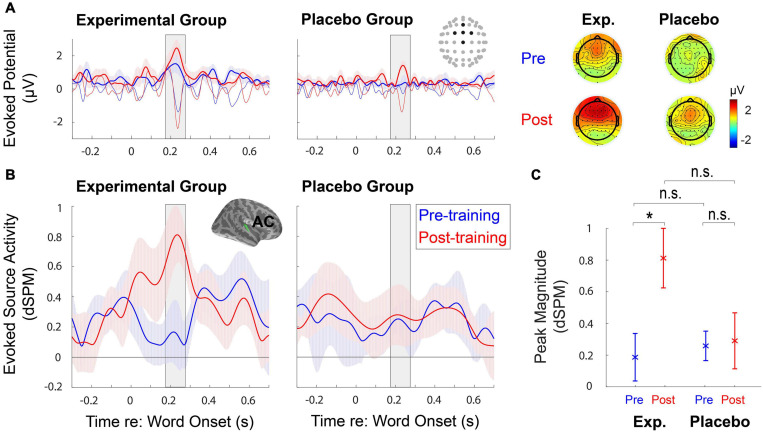
**(A)** The mean time courses of evoked potentials and topographies elicited to word-in-noise are obtained at the sensor-space level during pre- and post-training speech-in-noise tests. Thin lines indicate raw evoked potentials, while thick lines represent the temporal envelope of those evoked potentials. **(B)** The region-of-interest-based source analysis shows the mean time courses of the temporal envelope of evoked source activity, with ± 1 standard error of the mean (SEM), obtained at the right auditory cortex (AC) during pre- and post-training speech-in-noise tests. **(C)** The comparison of the mean (±1 SEM) peak magnitudes of the temporal envelopes. The peak magnitudes obtained at the right AC significantly differ before and after training in the Experimental Group. *Significant at *p* < 0.05, n.s., not significant.

The quality of EEG data was firstly checked by examining ERPs obtained at the sensor-space (Figure 5A). Clear auditory components (e.g., N1) found from the front-central channels allowed us to analyze ERPs at the source-space level further. Source-space data obtained from the right hemisphere (Figure 5B) showed that AC responses to *target* speech increased significantly after training (*F*_1,18_ = 4.78, *p* = 0.042); notably, the training effect only appeared in the Experimental Group (*t*_9_ = −3.16, adjusted *p* = 0.023), but not in the Placebo Group (*t*_9_ = −0.14, adjusted *p* = 1) (Figure 5C). The *F*-test results revealed no group effect (*F*_1,18_ = 1.94, *p* = 0.18); the *post hoc* analysis showed no significant difference in AC responses to target speech between two groups before (*t*_18_ = −0.41, adjusted *p* = 1) and after training (*t*_18_ = 2.024, adjusted *p* = 0.12) (Figure 5C). There existed no significant interaction between training and group (*F*_1,18_ = 3.89, *p* = 0.064). On the contrary, from the left hemisphere, the AC responses to *target* speech did not show significant main effects [training (*F*_1,18_ = 0.22, *p* = 0.65), and group (*F*_1,18_ = 0.32, *p* = 0.58)] and interaction between training and group (*F*_1,18_ = 0.33, *p* = 0.57). For AC responses to *ignored* speech (i.e., background babble noise), no significant main effects and interaction between training and group were revealed in the right hemisphere [training (*F*_1,18_ = 0.43, *p* = 0.52), group (*F*_1,18_ = 0.0057, *p* = 0.94), and interaction (*F*_1,18_ = 1.093, *p* = 0.31)] and in the left hemisphere [training (*F*_1,18_ = 0.94, *p* = 0.35), group (*F*_1,18_ = 1.74, *p* = 0.20), and interaction (*F*_1,18_ = 0.024, *p* = 0.88)].

## Discussion

Our training paradigm was designed to reinforce attentional modulation of auditory cortical evoked potentials by providing visual neurofeedback determined by an EEG-based attention decoder. After four sessions of training, the Experimental Group exhibited consistent improvement in the attentional modulation of the evoked responses to the training stimuli, whereas the Placebo Group did not show such an effect despite being exposed to near-identical auditory stimuli and repetitive selective attention task. This finding implies that the type of feedback is a critical factor in determining the efficacy of attention training. In addition, subjects in the Experimental Group showed enhanced neural encoding of target speech and improved performance during a post-training speech-in-noise task, indicating that the training effect could be transferred to untrained stimuli.

Better representation of target speech at AC after training may reflect an active sensory gain control for the Experimental Group (Hillyard et al., 1998). This is consistent with previous findings showing that attention could modulate the sound representation in AC and improve behavioral performance (Mesgarani and Chang, 2012; Carcea et al., 2017; Kim et al., 2021). Listeners’ attentive ability in noisy environments involves both the ability to fixate on a target and the ability to suppress distractors (Bidet-Caulet et al., 2010; Chait et al., 2010), which may independently account for individual differences in selective attention (Petersen et al., 2017; Schwartz and David, 2018). Our neurofeedback training tended only to facilitate enhanced responses to target speech in AC. Still, it did not lead to suppressed responses to ignored speech, indicating that AC responses to target speech were only trained by this training paradigm. Furthermore, only the right hemisphere data showed significant differences in AC responses to target speech. Given reported right-hemispheric dominance for auditory processing and attention (Alexander et al., 1996; Jemel et al., 2002; Stevens et al., 2006) and left-hemispheric dominance for language processing (Hickok et al., 2002; Tyler et al., 2011), the present study’s results may validate the effect of selective attention training while avoiding confounding factors related to language processing.

It should be noted that learning or training can facilitate the selective enhancement of neural response, which may develop over time and last longer, and improve speech perception (Froemke et al., 2013). The training effect found in the present study may develop and last over the weeks of training, resulting in improvement in speech perception, although the current study does not address how long the effect of training lasts after the neurofeedback training is over. Given the evidence of time-limited memory consolidation (Roesler and McGaugh, 2010), the training effect may not continue for the very long term. As stated above, the main difference between the Experimental Group’s training and the Placebo training was *how* the feedback reward was determined: by participants’ neural activity or behavioral performance. This difference in the feedback scheme led to a significant difference in how attentional modulation alters over time during training; the strength of attentional modulation averaged across Experimental Group subjects increased monotonously with repeated neurofeedback training, whereas it decreased in the Placebo Group. The Placebo Group subjects achieved near-perfect attentional performance throughout the training but without being motivated to strengthen their attentional modulation. This difference in training effect between groups may indicate a dissociation between attentional performance and attentional modulation of neural responses.

Similar to the reports by Whitton et al. (2014, 2017), the present study showed that the effect of neurofeedback training could be transferable to SiN performance, while the Placebo Group did not show such generalizability. The generalizability of the training effect observed in the present study may indicate that reinforcing attentional modulation of cortical responses could be one of the key neural strategies for the SiN understanding. Due to effective training that engages participants actively with a challenging task, the Experimental Group may have learned how to utilize the rhythmic structure of sound inputs from the training. Anecdotally, this was indeed a common strategy employed by the participants. Utilization of the rhythmic structure and patterns resulted in a better release from masking noise during our SiN task due to its fixed timing of noise and target onsets. Emerging evidence implicates that attention modulates AC representation by enhancing the neural “entrainment” to the predicted rhythm of speech streams (Golumbic et al., 2013) and that the sensitivity to “rhythm” may be the most prominent predictor of SiN performance among other aspects of musical abilities (Yates et al., 2019). In contrast, our Placebo training provided a primary task of detecting pitch oddballs from the attended stream. Feedback provided to the Placebo Group informed how accurate their oddball detection was, *not* how strong their attention was. Our results may indicate that the “indirect” primary task that does not provide a reward, shaped by the strength of attentional modulation, would not likely exhibit the generalizability of its training effect to the SiN task.

There are some limitations to this study. Due to requiring four training sessions per subject in each group, having ten people for two groups requires overall 80 sessions of visits that involve the EEG setup. Although the sample size of the current study can be justified by a power analysis based on the previously-reported effect size of perceptual training, the experiment may become underpowered in the number of subjects with the high variability in pre-test accuracy in the Placebo Group, which would likely be reduced with more subjects. Note that although the 4-week training was chosen based on the literature review and reached the significant training effect, we would not conclude that the number of sessions (i.e., one session per week) used in the present study was the most effective. A greater number of subjects and the validation of training interval and frequency are expected for future studies.

Furthermore, the decoding algorithm used for neurofeedback training did not achieve a very high level of accuracy (∼60%) in the present study, implying some trials provided inaccurate feedback. This level of accuracy raises two issues. First, was the quality of the proposed neurofeedback determination reliable enough to capture attentional modulation? Second, how close was the 60% accuracy to the optimal ratio of rewards for learning? Regarding the first issue, our decoding accuracy might have been underestimated since it has been derived based on the assumption that all subjects successfully paid attention to the target stream. However, realistically, subjects might have missed the visual cue or loosen their engagement to the task occasionally. The precise accuracy of the attention decoder can be derived only when you know subjects’ behavioral performance: how often subjects actually pay attention to the target stream. Indeed, in most previous studies (Choi et al., 2013; O’Sullivan et al., 2015; Teoh and Lalor, 2019), attention decoding has been calculated only based on correctly-answered trials. We could not obtain the number of correctly answered trials because we intentionally did not assign behavioral task to the Experimental Group. The attention decoder algorithm was near identical to the one used by Choi et al. (2013), which demonstrated ∼70% accuracy among correctly-attended trials. Although even 70% accuracy could be claimed insufficient, this level of decoder accuracy left room for improvement with repeated training, which was a positive factor for this study. Regarding the second issue (*the optimal ratio of rewards that facilitates learning*), previous learning tasks typically set reward contingencies at 70% [e.g., Bai et al. (2014); Correa et al. (2018)], which is not very high nor very low. A very high ratio (e.g., 90%) or very low rate (e.g., 10%) of rewarding may fail to motivate listeners to engage themselves in the learning process because subjects may under or overestimate their ability to successfully engage in the task. In that sense, ∼60% rewarding ratio was not very far from the ratio of reward proven effective by previous learning studies. However, we cannot argue how close to the *optimum* our decoder accuracy was. Future studies should seek optimal decoder accuracy and rewarding ratio for attention training.

In the present study, to maximize stream segregation, the competing streams in our attention training differed in their location, speaker identity, and tempo. Thus, it is unclear what acoustic cues mainly contribute to the training effect. Future studies should compare a range of acoustic cues to evaluate what is useful for selective attention training and investigate whether such cues are also available in a more general form of SiN task. Additionally, this study did not inform whether the training effect remains after the training period or becomes extinct, as reported by Whitton et al. (2017); a future study is expected to test the extinction of training effects. Lastly, given that peripheral hearing damage interferes with the cocktail-party listening that requires selective attention (Shinn-Cunningham and Best, 2008), future studies need to investigate this training effect in clinical populations (e.g., hearing aid or cochlear implant users).

## Data Availability Statement

The raw data supporting the conclusions of this article will be made available by the authors, without undue reservation.

## Ethics Statement

The studies involving human participants were reviewed and approved by University of Iowa Institutional Review Board. The patients/participants provided their written informed consent to participate in this study.

## Author Contributions

IC designed the experiments. SK and CE ran the experiments. SK and IC analyzed and interpreted data. All authors wrote the manuscript.

## Conflict of Interest

The authors declare that the research was conducted in the absence of any commercial or financial relationships that could be construed as a potential conflict of interest.
